# Incorporating Remote Electrical Neuromodulation (REN) Into Usual Care Reduces Acute Migraine Medication Use: An Open-Label Extension Study

**DOI:** 10.3389/fneur.2020.00226

**Published:** 2020-04-07

**Authors:** Michael J. Marmura, Tamar Lin, Dagan Harris, Alon Ironi, Noah L. Rosen

**Affiliations:** ^1^Jefferson Headache Center, Thomas Jefferson University, Philadelphia, PA, United States; ^2^Theranica Bio-Electronics Ltd., Netanya, Israel; ^3^Northwell Health, Great Neck, NY, United States

**Keywords:** remote electrical neuromodulation, migraine, medication overuse headache, conditioned pain modulation, neuromodulation, acute treatment

## Abstract

**Background:** A recent randomized controlled study showed that 66.7% (66/99) and 37.4% (37/99) of people undergoing remote electrical neuromodulation (REN), a novel non-pharmacological migraine treatment, achieve pain relief and pain freedom, respectively, at 2 h post-treatment. The participants who completed the 6-weeks double-blind phase of this study were offered to participate in an open-label extension (OLE) with an active REN device.

**Objective:** This study investigated the clinical use of REN, focusing on its potential in reducing the use of acute migraine medications.

**Methods:** The parent study for this open-label extension (OLE) was a randomized, double-blind, sham-controlled study of acute treatment conducted on 296 participants enrolled at 12 sites in the USA and Israel. This study included a run-in phase, in which migraine attacks were treated with usual care, and an 8-weeks double-blind treatment phase. One hundred sixty participants continued in an 8-weeks OLE phase in which they could incorporate a REN device into their usual care. Medication use rate (percentage of participants who treated their attacks only with REN and avoided medications in all their attacks) and pain outcomes at 2 h post-treatment were compared between the OLE and the run-in phase in a within-subject design.

**Results:** The analyses were performed on 117 participants with episodic migraine. During the OLE, 89.7% of the participants treated their attacks only with REN and avoided medications in all their attacks compared with 15.4% in the run-in phase (*p* < 0.0001). The rates of pain relief and pain-free in at least 50% of the treatments at 2 h post-treatment were comparable (pain relief: 58.1% in the run-in phase and 57.3% in the OLE, *p* = 0.999; pain-free: 23.1% in the run-in vs. 30.8% in the OLE, *p* = 0.175).

**Conclusions:** REN may reduce the use of acute migraine medications. Thus, incorporating REN into usual care may reduce the risk for medication overuse headache (MOH). Future studies should evaluate whether REN reduces the use of acute migraine medications in a population at risk for MOH.

## Introduction

Medication overuse headache (MOH) may occur in patients with a pre-existing disorder such as migraine as a consequence of frequent acute medication use with treatments such as triptans, ergots, barbiturates, or opiates as a complication of their underlying condition (1, 2). MOH manifests as an increase in the frequency and intensity of headaches and may lead to migraine chronification (1, 3). Migraine chronificiation may also result from ineffective acute treatment, obesity, depression, and stressful life events, although overuse of acute migraine medication is the most important risk factor for migraine chronification (4). The main approaches for MOH prevention include education, preventive therapies, restriction of monthly doses (5), and withdrawal of the overused medication(s) accompanied by or followed by prophylactic treatment (6, 7). High-frequency usage of acute medications such as triptans, ergots, barbiturates, anti-inflammatories, or opiates may cause adverse events (AEs) including gastrointestinal issues, renal or hepatic toxicities, dependency, and withdrawal, and interference with other medications and medical conditions.

There are several non-pharmacological interventions used for the treatment of headache, including behavioral techniques (e.g., biofeedback, cognitive behavioral therapy, and relaxation) and neuromodulation methods (e.g., external trigeminal nerve stimulation, non-invasive vagus nerve stimulation, and single-pulse transcranial magnetic stimulation). However, the limited access to behavioral treatments and the low adherence remain barriers for behavioral interventions (8), and the efficacy of current neuromodulation devices (9–11) appears inferior to that reported for migraine-specific pharmacological treatments (12).

Remote electrical neuromodulation (REN) is a novel acute migraine treatment (13), in which upper arm peripheral nerves (median and musculocutaneous) are stimulated to induce conditioned pain modulation—a descending endogenous analgesic mechanism in which sub-threshold conditioning stimulation inhibits pain in remote body regions (14). A recent randomized, double-blind, sham-controlled, multicenter study demonstrated that REN provides superior, clinically meaningful relief of migraine pain and complete pain freedom at 2 h post-treatment compared to sham stimulation. Active stimulation was more effective than sham stimulation in achieving pain relief (66.7 vs. 38.8%, *p* < 0.0001), pain freedom (37.4 vs. 18.4%, *p* = 0.003), and relief of most bothersome symptom (46.3 vs. 22.2%, *p* = 0.0008) at 2 h post-treatment. The pain relief and pain freedom superiorities of the active treatment were sustained for 48 h post-treatment (15). This study also demonstrated a low incidence of device-related AEs which was similar between the treatment groups (4.8 vs. 2.4%, *p* = 0.499). All device-related AEs, mainly sensations of warmth and redness of the treated arm/hand, were mild, resolved within 24 h, and did not require medical treatment (15).

The objective of this study was to evaluate the potential of REN in reducing acute medication use, which may in turn decrease the risk for MOH and shed light on the potential utilization of a non-pharmacological neuromodulation treatment as an alternative or supplement to usual care. Here we report the results of an open-label extension (OLE) study of people with episodic migraine completing a 6-weeks, double-blind, sham-controlled pivotal study of REN for acute treatment of migraine (15).

## Materials and Methods

### Study Design

The parent study for this OLE was a randomized, double-blind, sham-controlled study conducted at 12 sites (seven in the USA and five in Israel) on patients 18–75 years old who met the International Classification of Headache Disorders (3-beta) criteria (16) for episodic migraine. All procedures were approved by the institutional review boards at all participating sites. This trial is registered with ClinicalTrials.gov (NCT03361423).

Detailed descriptions of the study design and the stimulation properties of the REN device (Nerivio™, Theranica Bio-Electronics Ltd., Israel) have been reported elsewhere (15). Briefly, after enrollment, the participants were trained to use an electronic diary, and then they completed a 2- to 4-weeks run-in phase, during which the attacks were treated according to usual care and pain scores (none, mild, moderate, or severe) were recorded at baseline and 2 h post-treatment. Eligible participants were then randomized in a 1:1 ratio to either active or sham stimulation, in a double-blind manner. The participants treated their migraine attacks with the device for 4–6 weeks (double-blind treatment phase), and for each treated attack, they recorded the intensity of the headache at baseline, at 2 h, and at 48 h after the treatment in the electronic diary in the app. The participants were instructed to begin a treatment as soon as possible after the migraine symptoms began and always within 1 h of symptom onset. The participants who completed the double-blind treatment phase were eligible to continue in an 8-weeks open-label extension trial in which they could treat their migraine attacks with a REN device and/or with their usual acute care according to their preference. An optimal individual stimulation intensity level that was easily perceptible but not painful was determined for all participants. Pain scores (none, mild, moderate, or severe) were recorded at baseline and at 2 h post-treatment using the app. Throughout the phases of the study, medication use was also recorded in the app at 2 h and at 48 h after treatment.

### Participants

The eligibility criteria for enrollment in the parent study have been reported previously (15). Briefly, The participants had two to eight migraine headaches per month, ≤12 headache days per month, and were on either no or stable migraine preventive medications in the last 2 months prior to recruitment. To be eligible to continue in the OLE phase, the patients had to complete the double-blind treatment phase. The study protocol was reviewed and approved by the appropriate institutional review board for each site and was conducted according to Good Clinical Practice and the Declaration of Helsinki guidelines. Before undergoing any study procedures, the patients provided written informed consent.

Statistical power calculation was conducted prior to the study on the primary endpoint of pain relief at 2 h in the double-blind treatment phase. A sample size of 234 participants (117 per treatment arm) was determined to provide 80% power to demonstrate a statistical significance of 0.05 for the primary endpoint, assuming a sham pain relief rate of 32% and a therapeutic gain of 18%. The sample size of the OLE study was based on the available data.

### Data Analysis

Medication use patterns during the OLE phase (i.e., when an alternative non-pharmacological treatment was available to the participants) were compared to the rates in the run-in phase (i.e., usual care with medications) in a within-subject design that included the participants who, during the OLE, used the device for the treatment of at least one attack for which pain intensity at 2 h post-treatment was reported. The percentage of participants who treated their attacks only with REN and avoided medications in all their attacks was compared between the run-in phase and the OLE phase. To assess the clinical impact of device adoption, pain outcomes at 2 h post-treatment were also compared. The analyses focused on the intra-individual consistency of response across multiple attacks since demonstrating the consistency of a treatment is clinically important as it would indicate that a treatment can be relied on by the patients, which can improve adherence, reduce migraine-related disability and anxiety, and increase self-efficacy (17). This evaluation included pain relief at 2 h post-treatment (defined as improvement from severe or moderate pain to mild or none or from mild pain to none) in at least 50% of the treated attacks and pain-free at 2 h post-treatment (defined as improvement from mild, moderate, or severe pain to none) in at least 50% of the treated attacks. This approach to assess consistency has an important advantage of using all available data and included all participants in the analyses. All efficacy analyses were performed on non-recurrent migraine headaches, defined as headaches preceded by at least 48 headache-free hours. The use of rescue medication before the 2-h assessment was considered as treatment failure.

To assess the safety of the REN device, all AEs were classified in relation to their severity, duration, and possible causal relationship to the device. The primary safety variable was the proportion of patients reporting one or more device-related AEs. The safety analysis was conducted on all participants who treated at least one attack with the device during the OLE phase.

For continuous variables, mean and standard deviation (SD) are provided. For categorical variables, the number and percentage of patients in each category are provided. Medication use patterns and efficacy data were compared using McNemar's test. Between-subjects analyses were assessed using the chi-square test. All statistical tests were two-tailed, with statistical significance set at *p* < 0.05. No adjustments were made for multiple comparisons. All statistical analyses were performed using SPSS Statistics v20.0 (IBM Corporation). All authors had full access to all study data.

## Results

### Participants

**Figure 1:**
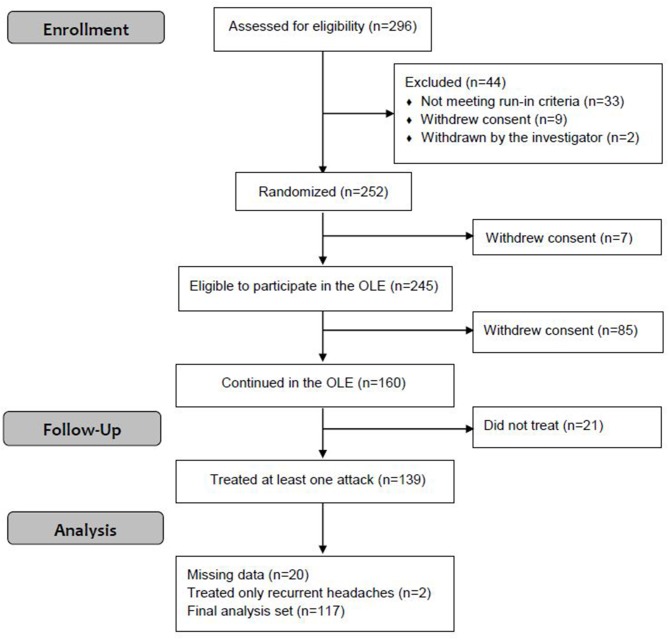
Enrollment of participants.

This OLE phase of the parent study was conducted from August 21, 2018 to January 29, 2019. Of the 296 participants enrolled in the parent study, 252 were randomized to active and sham groups; 245 participants were eligible to continue in the OLE [seven participants withdrew from the study (15)], of which 160 participants continued in the OLE. A total of 139 participants (86.9%) used the REN device at least once during the OLE, of which 117 participants had treated at least one non-recurrent migraine headache with REN for which pain intensity at 2 h post-treatment was reported (Figure 1). Of the 117 participants included in the analyses, 57 participants were in the active group during the double-blind treatment phase and 60 participants were in the sham group. Consistent with the typical clinical sample enrolled in a migraine study, the participants were predominantly female (78.1%) between the ages of 18 and 70 years old (Table 1). Clinical characteristics specific to migraine at baseline were consistent with the patient population with episodic migraine (Table 1). The demographic and clinical characteristics of the participants of the OLE are similar to that of the parent study (15).

**Table 1:** Demographic and clinical characteristics.

**Characteristic**	**All participants (*n* = 160)**
Age, years (SD)	44 (12.51)
Female, % (*n*/*N*)	78.1 (125/160)
Caucasian, % (*n*/*N*)	87.5 (140/160)
Triptan users, % (*n*/*N*)	45.62 (73/160)
Preventive medication use, % (*n*/*N*)	32.5 (52/160)
**Migraine With Aura, % (*****n*****/*****N*****)**	
Often	32.5 (52/160)
Rarely	20.6 (33/160)
None	46.9 (75/160)
**Most Bothersome Symptom, % (*****n*****/*****N***)^*^	
None	1.9 (3/160)
Nausea	28.7 (46/160)
Photophobia	49.4 (79/160)
Phonophobia	18.1 (29/160)

* *Two participants reported allodynia as most bothersome symptom (data not shown)*.

### Treated Migraine Attacks

**Figure 2:**
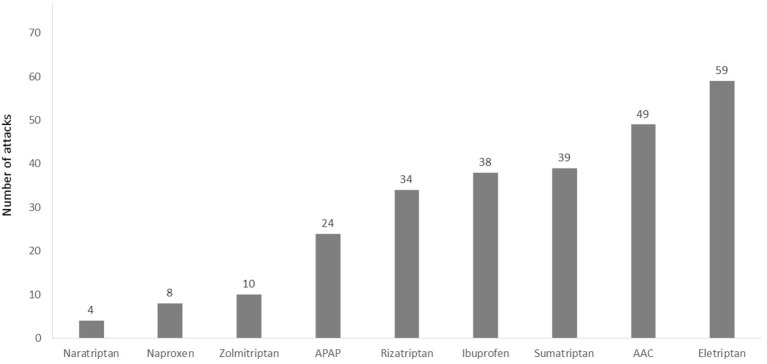
Number of attacks treated with different types of acute pharmacological treatments in the run-in phase. A total of 22 of the 404 attacks were excluded from this analysis since the medication type was not specified (participants reported “other” in medication type). AAC, aspirin, acetaminophen, and caffeine; APAP, acetaminophen.

In the run-in phase (in which the participants were not yet introduced to the REN device), a total of 404 non-recurrent migraine headaches were treated with usual care (which includes pharmacological treatments or no pharmacological treatment). The pain severity of the treated migraine attacks was mostly moderate (52.0%, 210/404) or mild (35.4%. 143/404). A total of 51/404 (12.6%) treated attacks were severe. Of the 404 reported attacks, the participants used acute medication for 287 attacks (71.0%); 49.5% of them (142/287) were treated with triptans. Figure 2 depicts the number of attacks treated with the different types of medication.

During the OLE, a total of 376 non-recurrent migraine headaches were treated with the device. There was no statistical difference in the mean treated attacks during the OLE between the participants who had experience with the active device in the double-blind treatment phase compared to those who did not (i.e., were in the sham group; *p* = 0.924), indicating that prior treatment experience did not affect the participants' willingness to continue in the open-label phase. The pain severity of the treated migraine attacks in the OLE was mostly mild (44.4%, 167/376) or moderate (42.3%, 159/376). A total of 50/376 (13.3%) of the treated attacks were severe. This distribution of baseline pain severity is similar to the baseline severity of all attacks reported in the run-in phase and in the double-blind treatment phase (15). Generally, the characteristics of the treated migraine headaches were comparable to those reported in previous migraine studies (11, 18, 19) and are consistent with the pain intensity characterization of the target population of the device (20).

### Medication Use Patterns

**Figure 3:**
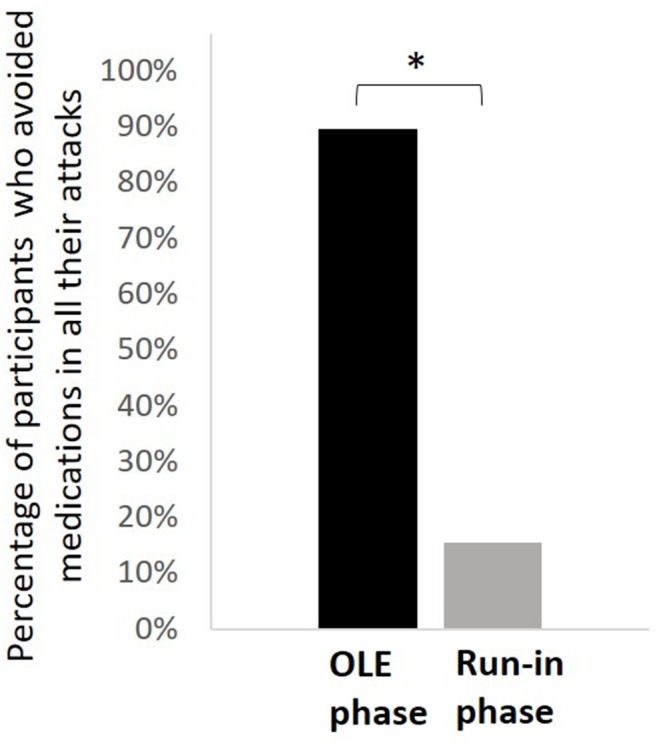
Comparison of medication use patterns. The percentage of participants who treated their attacks only with REN and avoided medications in all their reported attacks in the open-label extension phase (black) compared with the run-in phase (gray). *p < 0.0001.

The analyses were performed on 117 participants who had treated at least one migraine attack with REN for which pain intensity at 2 h post-treatment was reported. The mean reported attacks across subjects in the run-in phase was 3.44 ± 1.25 and 3.21 ± 2.27 in the OLE phase. During the OLE, 89.7% (105/117) of the participants treated their attacks only with REN and avoided medications in all their reported attacks compared with 15.4% (18/117) in the run-in phase (*p* < 0.0001; Figure 3). When REN was available for the acute treatment of the attacks, 73.5% (86/117) of the participants achieved ≥50% reduction in the number of attacks treated with medication and 42.7% (50/117) achieved 100% reduction in the number of attacks treated with medication.

### Clinical Pain Outcomes

**Figure 4:**
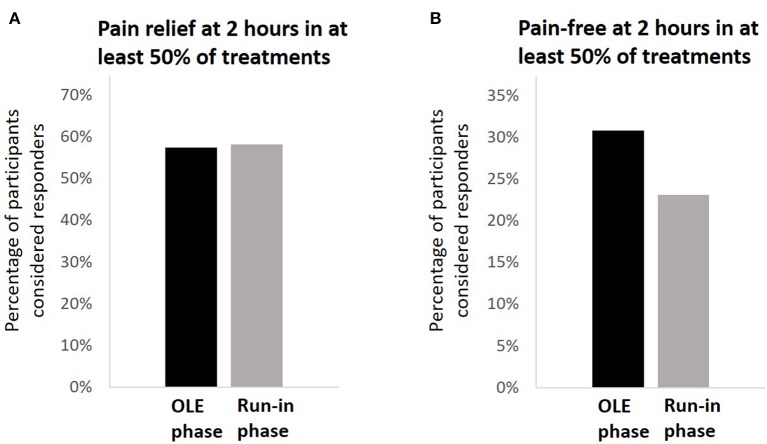
Comparison of clinical pain outcomes. **(A)** The percentage of participants achieving pain relief at 2 h post-treatment in a least 50% of their treatments in the open-label extension phase (black) compared with the run-in phase (gray). **(B)** The percentage of participants achieving pain freedom at 2 h post-treatment in a least 50% of their treatments in the open-label extension phase (black) compared with the run-in phase (gray).

The analyses were performed on the same population used for the medication use patterns assessment (*N* = 117). In the OLE, 57.3% (67/117) of the participants achieved pain relief at 2 h post-treatment in at least 50% of their attacks. compared with 58.1% (68/117) in the run-in phase (*p* = 0.999; Figure 4A). Among the 67 participants who achieved pain relief at 2 h post-treatment in at least 50% of their attacks, the mean pain relief rate was 77.3 ± 20.7%. A similar pattern of results was observed for pain-free response; in the OLE, 30.8% (36/117) of the participants achieved pain freedom at 2 h post-treatment in at least 50% of their attacks, compared with 23.1% (27/117) in the run-in phase (*p* = 0.175; Figure 4B). Pain relief and pain-free outcomes did not depend on whether the participants were in the active group or in the sham group in the double-blind treatment phase (*p* = 0.735 for pain relief; *p* = 0.099 for pain-free). The pain relief rates with REN were higher in people not using triptans (67.2%, 43/64) than in people who were using triptans (45.3%, 24/53; *p* = 0.017), but there was no significant difference in pain-free response between the groups [34.4% (22/64) in non-users vs. 26.4% (14/53) in triptan users; *p* = 0.353]. Across the 36 participants who achieved pain freedom at 2 h post-treatment in at least 50% of their attacks, the mean pain-free rate was 73.5 ± 20.9%.

### Safety

Safety analyses were performed on all 139 participants who used the device at least once during the OLE phase. Overall, 11 participants (7.9%) reported at least one AE and four participants (2.9%) reported device-related AEs. Device-related AEs included pain in the arm/hand, pain in the shoulders and neck, and muscle spasm. All device-related AEs were mild, resolved within 24 h, and did not require medical treatment. There were no serious device-related AEs and no unanticipated AE effects, and none of the participants withdrew from the study due to AEs. A summary of AEs that occurred during the OLE phase is shown in Table 2.

**Table 2:** Incidence of adverse events and device-related adverse events.

	**All participants who used the device at least once (*n* = 139)**
Patients reporting at least one adverse event, % (*n*/*N*)	7.9 (11/139)
Device-related adverse events, % (*n*/*N*)	2.9 (4/139)
Pain in the arm/hand, % (*n*/*N*)	1.4 (2/139)
Muscle spasms, % (*n*/*N*)	0.7 (1/139)
Neck and shoulder pain, % (*n*/*N*)	0.7 (1/139)

## Discussion

The results of this study demonstrate that REN holds the potential to reduce acute medication use in patients with migraine. The OLE phase was designed to resemble real-world clinical practice, in which the participants were provided with the opportunity to incorporate REN into their usual care for the acute treatment of migraine. The percentage of participants treating all their attacks with REN and avoiding medications during the OLE was very high−89.7%—and significantly improved when compared to the run-in phase (i.e., usual care with pharmacological treatments). There was no reduction in pain relief and pain-free rates associated with the incorporation of REN into usual care, with over 57% of the participants achieving pain relief at 2 h post-treatment in at least 50% of their treatments and over 30% of the participants achieving a pain-free outcome at 2 h post-treatment in at least 50% of their treatments. This indicates that the efficacy of REN is similar to usual care and that reducing medication use (and using the device instead) did not hinder the relief of migraine pain.

In the parent study, the subjects were required to refrain from using acute medication prior to REN. In this OLE, the participants were not instructed to avoid using acute medications with REN for the treatment of migraine attacks. Thus, the reduction in medication use patterns observed during the OLE may reflect the desire of the patients to adopt new non-pharmacological treatments that may replace their current usual care, which mainly relies on medications. This notion is also supported by the great proportion of participants (~87%) who used the device at least once, even though the study did not include this requirement. The acceptance of REN for the acute treatment of migraine suggests that REN may provide an alternative treatment for people with a great need for non-pharmacological treatments, such as patients with MOH undergoing detoxication. It also indicates that REN may overcome barriers to the adherence of headache behavioral treatments (8).

The reduction in medication use patterns achieved by incorporating REN into usual care has important clinical significance in the treatment of MOH due to frequent intake of pain medications. It is well-acknowledged that prevention of MOH/chronic migraine should be a primary goal when treating episodic migraine (21). The current approach for the prevention of MOH, which relies on education, preventive therapy, and limiting the monthly doses, requires to provide patients with an alternative treatment for symptoms relief, preferably non-pharmacological (5). It has been previously shown that cognitive behavioral therapy can prevent medication overuse in patients at risk (22). Many factors, such as poor coping strategies and lack of insight or motivation, time constraints, and cost or lack of qualified practitioners, negatively impact on adherence to non-pharmacological behavioral treatment (8). The results of the current study point to the high acceptance rates of REN and suggest that the advantage of REN is 2-fold: incorporating REN into usual care reduces medication use which may in turn reduce the risk for MOH, and it offers the patients an alternative non-pharmacological treatment with comparable clinical benefits.

The results of the current study indicate that REN treatment is effective and consistent. Pain relief and pain-free outcome at 2 h post-treatment in at least 50% of the attacks were achieved by 57.3 and 30.8% of the participants, respectively. Interestingly, the pain relief rates were lower in triptan users, but pain-free response did not depend on triptan use. Thus, it is safe to postulate that REN provides an alternative treatment for people who have been using triptans (23). Furthermore, the greater efficacy shown in non-users may support the choice of REN as an alternative to starting triptans for acute treatment. Consistent with a recent study (24), the results of this study also indicate that the efficacy of REN is non-inferior to usual care. Since in most attacks medications were avoided, it is safe to postulate that REN is superior to behavioral headache acute treatments, which typically supplement but do not replace medication treatments (25).

The results of the OLE also provide further support for the favorable safety profile demonstrated in the double-blind treatment phase of the study (15). The types and natures of the AEs and the very low incidence rates were comparable with previous observations and did not reveal any new safety concerns.

The current study has several limitations. First, this study reports on an 8-weeks OLE phase and did not include a direct observation on the development of MOH. Second, the study was conducted on people with episodic migraine and did not specifically focus on a population at risk for MOH or chronic migraine. However, most patients with MOH have an episodic headache history (21), suggesting that the results can be generalized. In addition, the rate of triptan use in the current study is higher than that of the general population (26), presumably due to recruitment at specialty centers. Yet these rates of triptan use reflect the rates observed in the population of adults with migraine with acute medication overuse (27), which has been associated with an increased risk of MOH. Third, there was a low rate of severe baseline pain intensity, presumably due to the early treatment. Finally, our dataset of usual care of multiple attacks included different pharmacological treatments (or no treatment) for a single person, which decreases its scientific purity; however, this intra-individual variability encompasses real-life migraine management, varying across attacks within the same patient and thus empowering our findings. Indeed performing the analyses over multiple attacks in a within-subject design is one of the strengths of this study, enabling to reduce the bias of novelty and signifying the consistency of the effect.

## Conclusions

Incorporating REN into usual care may have a positive impact on migraine management by reducing the reliance on acute medications. These results suggest that REN may be useful to prevent medication overuse headache and/or the transition from episodic to chronic migraine. Future studies should evaluate whether REN reduces the use of acute migraine medications in a population at risk for MOH.

## Data Availability Statement

All datasets generated for this study are included in the article/supplementary material.

## Ethics Statement

The studies involving human participants were reviewed and approved by the appropriate institutional review board for each site and were conducted according to Good Clinical Practice and the Declaration of Helsinki guidelines. The patients/participants provided their written informed consent to participate in this study.

## Author Contributions

AI and DH conceived and designed the study. MM, DH, and NR acquired the data. TL, DH, and AI analyzed the data. MM and TL wrote the paper. All authors approved the final manuscript.

### Conflict of Interest

MM has received compensation for consultation from Teva, Antres Pharma, Alder, Lilly, Promius, Supernus, Amgen/Novartis, Theranica, and GammaCore. He has participated in speaker bureaus for GammaCore, Eli Lilly, and Amgen/Novartis. He has received research support for serving as principal investigator from Teva, Allergan, and Theranica. TL, DH, and AI are employees of Theranica. NR has received compensation for consultation from Allergan, Amgen/ Novartis, Biohaven, Eli Lilly, Promius, Supernus, and Teva. He has participated in a speaker bureau for Allergan. He has received research support as an investigator from Allergan, Eli Lilly, Theranica, and Axon Optics. He has received compensation as an associate editor from Springer.
